# Gene Properties and Chromatin State Influence the Accumulation of Transposable Elements in Genes

**DOI:** 10.1371/journal.pone.0030158

**Published:** 2012-01-17

**Authors:** Ying Zhang, Dixie L. Mager

**Affiliations:** 1 Terry Fox Laboratory, British Columbia Cancer Agency, Vancouver, British Columbia, Canada; 2 Department of Medical Genetics, University of British Columbia, Vancouver, British Columbia, Canada; Texas A&M University, United States of America

## Abstract

Transposable elements (TEs) are mobile DNA sequences found in the genomes of almost all species. By measuring the normalized coverage of TE sequences within genes, we identified sets of genes with conserved extremes of high/low TE density in the genomes of human, mouse and cow and denoted them as ‘shared upper/lower outliers (SUOs/SLOs)’. By comparing these outlier genes to the genomic background, we show that a large proportion of SUOs are involved in metabolic pathways and tend to be mammal-specific, whereas many SLOs are related to developmental processes and have more ancient origins. Furthermore, the proportions of different types of TEs within human and mouse orthologous SUOs showed high similarity, even though most detectable TEs in these two genomes inserted after their divergence. Interestingly, our computational analysis of polymerase-II (Pol-II) occupancy at gene promoters in different mouse tissues showed that 60% of tissue-specific SUOs show strong Pol-II binding only in embryonic stem cells (ESCs), a proportion significantly higher than the genomic background (37%). In addition, our analysis of histone marks such as H3K4me3 and H3K27me3 in mouse ESCs also suggest a strong association between TE-rich genes and open-chromatin at promoters. Finally, two independent whole-transcriptome datasets show a positive association between TE density and gene expression level in ESCs. While this study focuses on genes with extreme TE densities, the above results clearly show that the probability of TE accumulation/fixation in mammalian genes is not random and is likely associated with different factors/gene properties and, most importantly, an association between the TE insertion/fixation rate and gene activity status in ES cells.

## Introduction

Since the identification of transposable elements (TEs) in maize by Barbara McClintock more than half century ago [1], these sequences have been found in almost all organisms. In mammals, four major types of TEs have been identified, namely long interspersed elements (LINEs), short interspersed elements (SINEs), long terminal repeat (LTR) retroelements (including endogenous retroviruses (ERVs)), and DNA transposons [2]. Each type of these TEs can also be divided into several families/clades, which differ in their age, DNA sequence, as well as the genomic distribution in the host organisms. For example, in both humans and mice, the majority of LINEs (83% for human and 98% for mouse) are the L1 (LINE-1) elements, which are relatively young with some still active today [3], [4]. However, the genomic composition of LINEs can be significantly different in some other mammals such as the cow, where only about half such elements are L1s with the remaining LINEs primarily belonging to the RTE (retrotransposable element) clade [5], which is virtually missing in the other two species.

While the vast majority of fixed TEs are likely neutral, they can also be either deleterious or beneficial depending on the individual case. For example, in humans some copies of L1s and Alus (SINE) are currently active, and genetic disorders linked to *de novo* insertions of these elements have been reported (e.g. Apert syndrome [6], granulomatous disease [7]). Although LTR elements in humans are generally inactive, they are the major type of mutagenic TE in mice and cause ∼10% of mouse germ-line mutations [8]. On the other hand, an increasing number of studies have also shown that TEs can, sometimes, be utilized to fine tune or alter the regulation of host genes [9], [10], [11], with one fascinating example of the ERV-derived mammalian Syncytin genes that play crucial roles in placenta formation [12], [13]. Indeed, it is this ‘dual-role’ characteristic of TEs that likely has helped to shape the genomic landscapes of a wide range of host species, making TEs an important source of genetic diversity in evolution [14], [15], [16], [17].

During the last decade, increasing availability of whole genome sequences has revealed insights into the relationships of TEs and genes. For example, it has been well studied and widely accepted that the genome-wide distribution of TEs is far from random, which is likely primarily due to the long-term effects of natural selection [18], [19], [20], [21], [22], [23]. While most such work has focused on identifying properties of TEs that might contribute to their genomic distribution (e.g. insertional orientation, local G/C content preference, etc.), we, on the other hand, were particularly interested in evaluating the host-TE relationship by examining properties of the genes in which they reside. Indeed, several studies of intronic TE density and gene function have shown that TE-poor genes are usually linked to developmental processes while TE-rich genes tend to be involved in metabolic pathways [24], [25], [26]. Moreover, TE density in genes is reportedly associated with gene expression patterns [25], [26], [27]. Indeed, a study of TE accumulation in *Drosophila Melanogaster* euchromatin examined TE density differences between soma- vs. germline-expressed genes [28] and found a higher TE insertion rate for the latter. Interestingly, Kunarso et al. recently also reported that TEs have impacted the core regulatory networks of both human and mouse embryonic stem cells (ESCs) by being involved in ES-specific transcription factor binding and gene regulation [29].

While providing evidence that multiple factors correlate with TE accumulation/fixation rate in genes, previous studies mentioned above are limited in the following aspects. First, when identifying TE-poor and TE-rich genes, TE density conservation among orthologous genes in multiple species was not considered. Given the extremely low probability of having similar numbers of independent TE insertions in orthologous genes among multiple species by chance, an enrichment of TE-rich/poor genes shared across species may lead to new insights of TE-host relations. Second, while a study addressed the relationship between TE distribution and gene expression in germline/early embryo tissues [28], it was focused on invertebrates and was only based on indirect measurement of chromatin status such as EST abundance and microarray expression data. To address these limitations, we examined TE densities in genes of three mammalian species, namely human, mouse and cow, which are sufficiently diverged that most recognizable TE insertions are independent among these genomes [5], [19]. Additionally, since more than half of TEs in cows belong to ruminant-specific TE families that do not even exist in humans and mice [5], it provided higher confidence when evaluating possible factors that may contribute to the extreme density of TEs. Here we examined various properties of the TE-rich/poor genes shared across the above three species, including gene function, conservation, tissue-specificity of gene expression and histone modification profiles in mouse ESCs. Our results indicate that TE distributions in genes have been determined by or correlate with multiple properties of host genes, which in turn reflects the influence of TEs on shaping the landscape of host genomes.

## Results and Discussion

### Determining sets of orthologous genes with the same extreme of TE densities

Although ∼90% of human RefSeq genes contain sequences derived from TEs (mostly in introns), the coverage of TEs in each gene can be quite different. To understand if this difference is completely random, we firstly identified genes that are ‘unusually’ enriched or depleted of TE sequences in three mammalian species, namely human, mouse and cow. According to the initial sequencing and comparative analyses of the mouse genome [19], more than half of all human TEs are lineage-specific elements inserted after the human-mouse divergence and 87% of all TEs recognizable in the mouse are lineage-specific, probably due to higher deletion/mutation rates in the mouse. The whole-genome sequencing of taurine cattle also revealed that at least 58% of TEs in the cow belong to ruminant-specific repeat families [5]. These data show that the majority of TEs detectable in the above species today are independent insertions introduced after their divergence. To find outlier genes enriched/depleted of TEs in each species, we downloaded the RepeatMasker annotation of TEs from the UCSC Genome Browser (http://genome.ucsc.edu) and calculated the TE density of each gene by taking the ratio of TE coverage and gene size. Because most small genes contain few TE sequences simply due to the limitation of their size (Figure S1; see Materials and Methods), we restricted our analyses to genes larger than 10 kb.

Next, we sought outlier genes using ‘top 10%’ as an optimized cutoff threshold for genes with the highest/lowest TE densities (see Materials and Methods for details about optimization). Since TE gene fraction (i.e. TE density in genes) is linked to the gene length [30], we decided to control our TE density outlier analysis by gene size. Moreover, in our previous study of TE underrepresentation zones (U-zones) in mammalian gene introns, we found a significant decrease of TE density near exons [23], suggesting that exon density is also a potential factor limiting the coverage of TEs in genes (i.e. genes with higher exon density are expected to have less TEs). Although exon density and gene size are not independent (Figure S2), we still controlled our outlier analysis for both factors (Figure S3; see Materials and Methods) due to their moderate association strength (*r* = 0.688, *r* is the correlation coefficient for gene size vs. 1/exon density). Finally, by intersecting our datasets from the three species, those genes identified as ‘shared outliers’ were selected for further analysis because of the low probability that their outlier status is due to random chance (single-side frequency < 0.0005). In total, we found 84 shared upper-outlier genes (SUOs) and 189 shared lower-outlier genes (SLOs) (Table S1), the numbers of which are both much higher than expected by chance (p<2.2e-16 for both SUOs and SLOs, proportion equality test).

### Chromosomal distribution and type composition of TEs in shared outlier genes

Before performing any functional analysis of the SUO/SLO genes identified above, we were curious to see if there is any distributional bias at the chromosome level. To answer this question, we first plotted physical locations of both SUOs and SLOs along each human chromosome. As shown in Figure S4, no apparent enrichment was found on certain chromosome(s) for both types of shared-outlier genes except a disproportionally high amount of SUOs on chromosome 19. When we counted the total SUOs and SLOs separately for each chromosome and produced scatter-plots according to chromosome size (Figure S5), chromosome 19 stood out as an outlier with a relatively short size (2% of the human genome) but encoding 13 of 84 (15.5%) SUO genes. Since human chromosome 19 is gene-rich compared with all other chromosomes [18], we wondered whether the majority of TEs in SUOs are SINE elements, which (especially Alus) have been shown as highly enriched near genes and GC-rich regions [18], [21]. Indeed, further examination revealed that 53 of 84 (61%) SUOs contain more than 50% TEs as SINEs, including all of the 13 SUOs located on chromosome 19. On the contrary, LINEs are known to associate with AT-rich/gene-poor regions [18], [21] and are overrepresented in gene poor chromosomes such as chromosome X [21], [31]. Interestingly, while only three SUOs are on chromosome X, two of them contain more than 50% TEs as LINEs. These results suggest that the distribution of SUOs is not random and likely associated with the TE composition and the overall G+C content/gene density of each chromosome.

**Figure 1:**
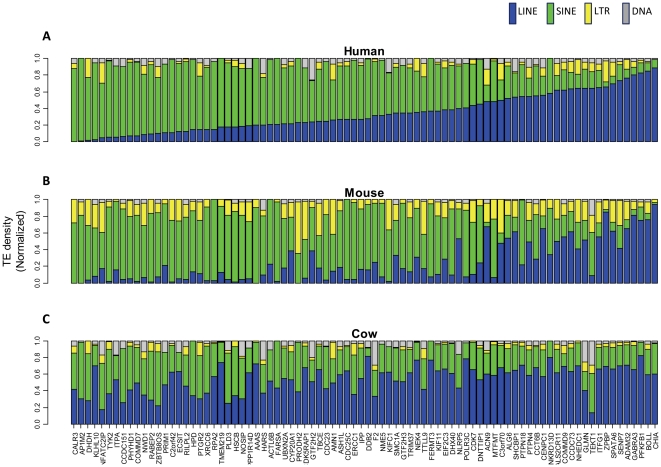
TE composition patterns of SUO genes. Patterns for human, mouse and cow are shown in (A), (B) and (C), respectively. Relative proportions taken by different TE classes for each SUO gene are shown as a stacked bar, with the color scheme for the four major TE classes indicated at the top. Genes are arranged in the same order for all three species. Gene names at the bottom are from the annotation of human RefSeq genes.

Next, we wondered if orthologous SUOs in different species contain the same types of TEs at similar percentages. When we examined the TE composition of the 84 SUOs, we found similar patterns for most of them between human and mouse (Figure 1A, B), but not for cow (Figure 1C). To further quantitatively measure the correlation of TE compositions between human and mouse SUOs, we calculated the proportion contributed by each of the four major TE types (i.e. the normalized TE composition) and the corresponding correlation coefficient (r). As shown in Figure S6, the proportion of LINE and SINE elements are highly correlated between human and mouse (r = 0.827 for LINEs; r = 0.836 for SINEs), but only poor correlations were found for ERV and DNA elements (r = 0.408 for ERVs; r = 0.267 for DNA elements). Furthermore, since the genomic density of LINEs or SINEs is highly associated with local G+C content [18], [21], [32], we calculated for all SUOs the correlation coefficient between the percentage of LINEs/SINEs and the local G+C content. As shown in Figure S7, a strong correlation to G+C content is found for both TE types (r = −0.702 for LINEs and r = 0.775 for SINEs), suggesting that it is a major factor determining the insertion/fixation probability of different TE types.

However, the same analysis in the cow genome showed much less tendency for similar TE composition patterns in SUOs compared with the human or mouse (Figure 1C). We reasoned that this is likely due to the large amount of TEs in the cow that are extremely rare in the other two species [5]. For example, almost half of the LINE elements in the cow belong to the RTE clade, which is also found in many other species including reptiles, insects and nematodes but absent from most mammals including primates and rodents [5], [33], [34]. Moreover, while most of the remaining LINEs are L1 elements (which is the most abundant LINE family in human and mouse), only 60% of them belong to subfamilies present in humans [34]. More dramatically, greater than 92% of Bovine SINEs are not found in both human and mouse and, unlike the major SINE families such as Alu in humans and B1 in mice, SINE elements in cows are either derived from tRNAs or truncated LINEs instead of 7SL RNAs [33]. Based on this unique genomic composition of TEs, it is not surprising to see a distinct composition pattern of TEs in cow SUOs. Indeed, a previous study has reported that L1s in the cow show little correlation with local G+C content, while RTE LINEs and most SINEs are both negatively associated with the density of local G+C [34]. This feature is apparently different from the case of human and mouse, in which LINEs (mostly L1) are negatively correlated with local G+C content while SINEs (mostly Alu/B1) show the opposite trend [18], [21], [32].

### Extreme TE density is associated with the function and conservation level of genes

In a pioneering study of Alu SINE distribution in genes based on sequence data of human chromosomes 21 and 22, Grover et al. reported an enrichment of Alu elements in genes involved in metabolic pathways and signaling and transport processes but a depletion from genes coding for information pathways and structural proteins [24]. The authors also postulated that both positive and negative selection forces had been involved in shaping the current distribution of Alus in human genes. In a different study on transposon-free regions (TFRs) in mammals [35], the authors revealed almost 1000 genomic regions larger than 10 kb that are completely depleted of TEs. Although over 90% of the bases covered by these regions are non-coding, genes within them are significantly associated with developmental functions, suggesting that these regions are largely unable to accept or tolerate TE insertions. While the above and some other genome-wide studies [25], [26] showed an association between TE density and specific gene functions, we wanted to determine if such trends exist for the outlier genes shared among multiple mammalian species after excluding the potential confounding effects of exon density and gene size. We examined our SUO/SLO gene lists derived from the three mammalian species mentioned above using BiNGO [36], which is a Gene Ontology (GO) tool that can be used to identify statistically significant over-/under-representation of certain gene functions for a given gene set compared to the genomic background. In accordance with previous studies of non-shared TE density outlier genes, we observed significant enrichment of genes involved in developmental processes for SLOs and enrichment of genes involved in metabolic pathways and DNA repair for SUOs (Table S2). Since these orthologous genes show similar extremes of TE density in several species and have been controlled by their size and exon density, our observation firmly supports the hypothesis that the density of TEs in genes is not random and is evidently associated with specific gene functions.

**Figure 2:**
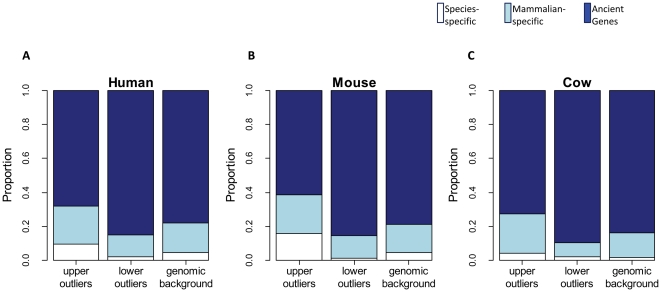
TE density and conservation level of outlier genes. Patterns for human, mouse and cow are shown in (A), (B) and (C), respectively. Proportions corresponding to different gene conservation levels for each gene set are shown as a stacked bar, with the color scheme for species-specific, mammalian-specific and ancient genes indicated at the top.

Intrigued by the possible association between TE density and functional ‘importance’ of genes, the next gene property we examined was the phylogenetic conservation level. In a recent study conducted by Mortada et al., the authors applied a comprehensive analysis of TE-free vs. TE-rich genes based on the difference of selection pressure ratio (Ka/Ks) among four primate species [25] but could not find significant support for the association between TE density and gene conservation level. The authors proposed that the phylogenetic distances between the four primates they examined might be too close and, indeed, when they looked at the selection pressure ratio between human and mouse, evidence was found that TE-free genes tend to be more conserved than TE-rich genes. To further test this hypothesis, here we defined three levels of gene conservation according to the HomoloGene database (release 63; http://www.ncbi.nlm.nih.gov/homologene): species-specific, mammalian-specific, and ancient genes (see Materials and Methods). In order to include species-specific genes, we expanded our analysis to all outlier genes including both shared and non-shared among human, mouse and cow, and obtained consistent results for all three mammalian species that we examined. In accordance with the results of Mortada et al. [25], here we found clear evidence showing a higher proportion of species-specific genes among SUOs and a higher proportion of ancient genes in SLOs (Figure 2). These findings show that TE-poor genes are more likely to be conserved among distantly related species, while genes extremely rich in TEs show an opposite trend. This suggests that TE insertions within highly conserved genes have generally been selected against due to their detrimental effects on these genes. On the other hand, the emergence of non-conserved, species-specific genes has increased the genetic variation of the host population, and our data supports the idea that TEs may have contributed to or even played an important role in creating such genetic diversity during evolution [14], [37].

Lastly, we compared the average TE ages between SUO and SLO genes in human and revealed a highly significant difference (p<2.2e-16, Wilcoxon Rank Sum test), with the former at 16.1% and the latter at 21.3% sequence divergence from their consensus sequences (For reference, 16–18% of unconstrained nucleotides have been substituted since the split of primates from other mammalian orders [32]). To evaluate whether such a difference in TE age was a reflection of a possible age difference between SUO and SLO genes, we randomly selected 200 mammalian-specific genes and another 200 ancient genes conserved also in fish or invertebrates and compared their ages (Materials and Methods). Interestingly, although the age of genes in the two random control groups is apparently different, the average age of TEs is about the same (19.7% vs. 19.1% divergence; p = 0.1367, Wilcoxon Rank Sum test). Based on the fact that the pattern of TE distributions is a combined effect of both initial TE integration patterns and outcomes of selection/genetic drift and thus may change through evolution [21], we postulate that TEs in SLOs may show more features of selection outcomes due to a longer evolutionary period, while the high TE density of SUOs may partially result from the fact that young TEs in such genes are still subject to natural lost or purifying selection.

### Binding of Polymerase II at gene promoters reveals association between TE-content and tissue-specificity of genes

Recently, Jjingo et al. reported that both higher TE density and larger gene size generally associates with less tissue-specificity of gene expression [27]. To determine which factor is more important to the tissue-specificity of genes, the authors applied multiple regression analyses and found a higher contribution of TE density (66%) than gene size (53%). While the above results provided important insights into the relationship between TE density and gene expression, the confounding effects of stochastic TE integration in a single species and gene structure constraints greatly impair the accurate evaluation of such relations. To overcome this problem, we directly compared polymerase-II (Pol-II, and more specifically, the Polr2a subunit of Pol-II) binding states between our SUO and SLO gene groups, which were controlled for both exon density and gene size.

**Figure 3:**
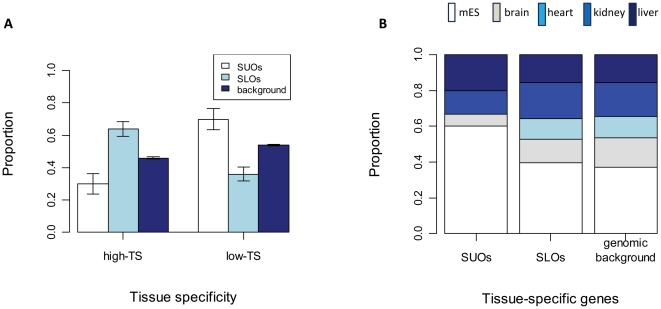
Tissue-specificity of Polr2a binding at outlier genes. (A) Associations between TE density and tissue-specificity of Polr2a binding. All gene sets are divided into two categories according to the Polr2a binding pattern of genes: the high tissue-specific (high-TS) and the low tissue-specific (low-TS). The proportions corresponding to the above two categories are shown for SUOs, SLOs and the genomic background as side-by-side bars. Error bars are standard errors derived from the total number of genes (sample size) in each gene set. (B) Tissue-type composition of SUOs/SLOs associated with strong Polr2a binding in only one tissue. For each gene set, the proportion taken by each tissue type is shown with corresponding color in a stacked bar. The ‘genomic background’ was calculated based on all mouse genes > 10 kb that show strong Polr2a binding in only one tissue. The color scheme for different tissue types is shown at the top.

To obtain accurate information regarding the tissue-specificity of genes, we downloaded the ChIP-chip genome-wide RNA Polr2a binding maps of active promoters in mouse embryonic stem cells (ESCs) and adult organs (brain, heart, kidney, liver) [38]. According to the tissue-specificity (TS) values as described by the authors, we classified our SUO and SLO genes into two categories, high-TS and low-TS (see Materials and Methods). As a control group, all genes larger than 10 kb in the mouse genome were similarly categorized and the proportions were compared with that of SUOs and SLOs. As shown in Figure 3A, our results reveal that SUO genes exhibit significantly lower tissue-specificity compared with the genomic background (30% vs. 46%; p = 0.0338), and vice versa for SLOs (64% vs. 46%; p = 0.0001), which is in accordance with previous findings [30].

Next, we were curious about whether there is any difference between tissue-specific SUOs and SLOs in terms of tissue-type composition. In other words, for those outlier genes expressed only in one tissue, it would be interesting to know whether specific tissue-types are significantly enriched. Using the same data set as above, we examined the tissue-type composition of all SUOs and SLOs that show strong Polr2a binding in only one tissue type (Materials and Methods). Despite identifying only 15 SUO genes satisfying our criteria, nine of these are observed in ESCs, a proportion (60%) much higher than either SLOs (40%) or the genomic background (37%) (Figure 3B). In order to gain more statistical power, we expanded our analysis to all mouse outlier genes including both shared and non-shared and, as shown in Figure S8, observed 89 ESC-specific upper outliers among a total of 181 upper outlier genes (49%), which is significantly higher than the genomic background level (37%; p = 0.00146). More interestingly, after expanding our analysis from shared-only to all outlier genes, we also observed a significant depletion of ESC-specific genes among tissue-specific lower-outliers (93 out of 367, or 25%; p = 1.406e-05). Based on the above observations and the fact that only TE insertions occurring in the germline and early embryonic cells could be inherited by the next generation, we propose that actively transcribed genes (presumably with an open chromatin state) are prone to TE integrations, which could lead to a faster accumulation of TEs in such genes during evolution.

### Histone marks at promoters confirm the overall open status of SUO genes in ESCs

**Figure 4:**
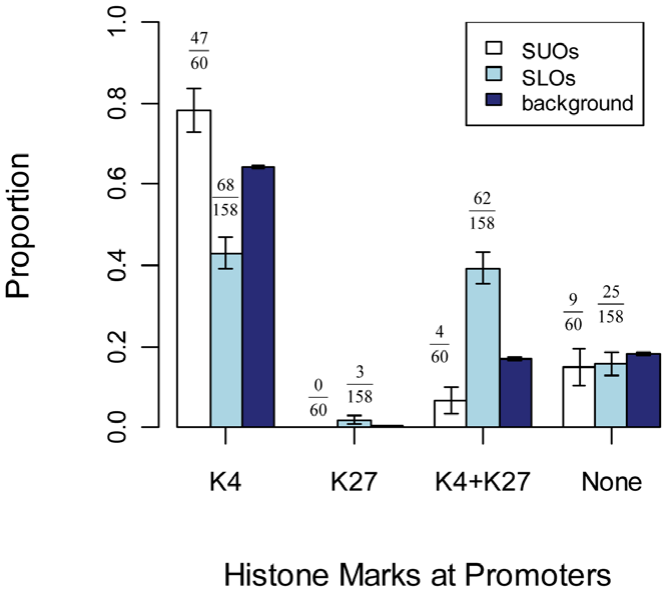
Histone marks at promoters of shared outlier genes. The proportions of genes associated with different histone marks are shown for SUOs, SLOs and the genomic background as side-by-side bars. Error bars are standard errors derived from the total number of genes (sample size) in each gene set.

To investigate our hypothesis that the chromatin regions of upper outlier genes are generally open in ES cells, we examined histone modification marks at gene promoters in mouse ESCs. In 2007, Mikkelsen et al. used ChIP-seq to generate genome-wide maps of various histone modification marks including the open-chromatin mark histone H3 lysine 4 trimethylation (H3K4me3) and the condensed-chromatin mark histone H3 lysine 27 trimethylation (H3K27me3) in mouse ESCs [39]. Although such histone modification data were largely limited to gene promoters in this study, these authors showed that both H3K4me3 and H3K27me3 can effectively discriminate genes that are expressed, poised for expression, or stably repressed, and therefore reflect both chromatin and transcriptional state. We used this dataset to evaluate the general chromatin status of both SUO and SLO genes in mouse ES cells (Figure 4). Intriguingly, our results revealed a significant enrichment of the open-chromatin mark H3K4me3 at promoters of SUOs (78%) but a depletion at SLO promoters (43%) compared with the genomic background (64%; p = 0.0333 and p = 5.653e-08 for SUOs and SLOs, respectively), indicating a general open state of SUOs and closed state of SLOs in mouse ESCs. When we looked at the closed-chromatin mark H3K27me3, we observed the opposite trend as for H3K4me3: SLOs are more associated with H3K27me3 (1.9%) than the genomic background (0.5%; p = 0.0766, equality proportion test) but there were no SUOs in this category (0/60 hit for H3K27me3). However, while the above observation is intriguing, there is unavoidably a lack of statistical significance due to the limited total number of SUOs and SLOs. On the other hand, when we examined the enrichment of the bivalent mark H3K4me3+H3K27me3, SLOs showed a much higher enrichment than the genomic background (39% vs. 17%; p = 5.591e-13) whereas SUOs showed the opposite effect (6.7% vs. 17%; p = 0.05). Since it has been shown that the H3K4me3+H3K27me3 bivalent mark is strongly associated with the ‘poised state’ of developmental genes that are temporarily repressed in ES cells but will become activated upon differentiation [40], the above result supports our earlier observation that many SLO genes are involved in essential developmental processes and, consequently, are depleted of TE insertions. Finally, for shared outlier genes that are not linked to either H3K4me3 or H3K27me3, no significant differences were detected when compared to the genomic background. The same analysis expanded to both shared and non-shared outlier genes showed very similar results, which are illustrated in Figure S9.

### Gene function and chromatin status both contribute to the low TE density of SLO genes

**Figure 5:**
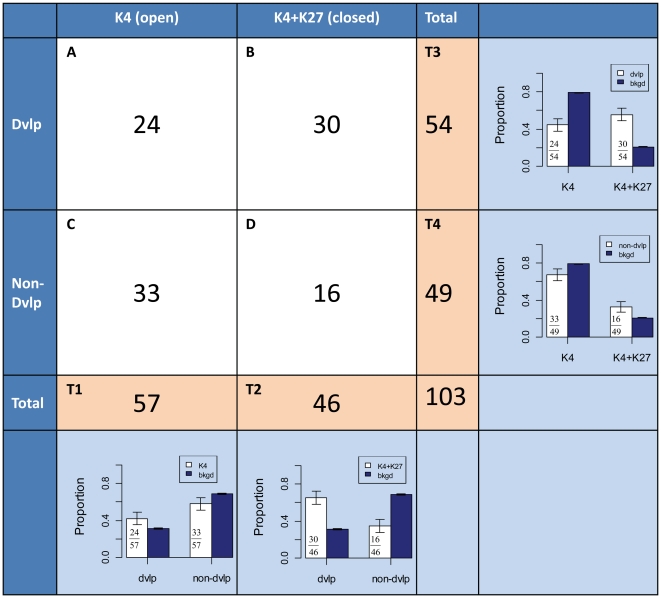
Effect-evaluation matrix for gene function and chromatin status of SLOs. As shown in the inner 2x2 matrix (white area), SLOs are divided into four groups according to their associated histone mark and gene function. ‘K4’ stands for H3K4me3; ‘K4+K27” stands for H3K4me3 + H3K27me3; ‘Dvlp’ stands for developmental gene; ‘Non-Dvlp’ stands for non-developmental gene. Bar plots in the most right column show the proportion of SLOs associated with different histone marks compared with the genomic background when gene function is controlled. Bar plots in the bottom row show the proportion of SLOs associated with different gene functions compared with the genomic background when chromatin status is controlled. Error bars are standard errors derived from the total number of genes (sample size) in each gene set.

Although the above evaluation of chromatin status at SUO/SLO genes using genome-wide histone modification maps clearly shows a correlation between the density of TEs and chromatin status in ES cells, such observations may be explained by at least two possible underlying mechanisms. First, TEs may be more likely to insert into genes with open chromatin, leading to an accumulation of TE sequences in genes active in ESCs. Alternatively, the strong association between low TE density and poised/inactive genes in ESCs could also be explained by the essential roles of these genes in cell differentiation and thus limited tolerance to TE residence. While the two mechanisms are not mutually exclusive, we were curious to separately evaluate the contributions of chromatin status and gene function. First, we classified all SLO genes depending on whether or not they are under the GO term of developmental processes. Then, we checked the SLOs associated with either H3K4me3-only (open state) or H3K4me3+H3K27me3 bivalent mark (poised state) and calculated the proportion of genes involved in developmental processes in each set (due to the limited number of SLOs associated with the H3K27me3-only mark, we did not include such analysis in this study). Interestingly, as seen as section A in Figure 5, 42% (24 out of 57) SLOs associated with an open chromatin state in ESCs were developmental genes, which is higher (but not statistically significant) than the whole-genome background (31%; p = 0.1). More dramatically, the analysis of SLOs associated with the H3K4me3+H3K27me3 bivalent mark showed 65% (30 out of 46) were developmental genes (section B in Figure 5), which is two fold more than the genomic background (p = 1.470e-06). Notably, the predicted chromatin status was not a variable in both analyses, confirming that gene function definitely plays a role in terms of influencing the density of TEs in genes. Moreover, the higher proportion of genes involved in developmental processes for SLOs associated with the H3K4me3+H3K27me3 bivalent mark (65%; B/T2 in Figure 5) compared with SLOs associated only with H3K4me3 (42%; A/T1 in Figure 5) also implies an effect of chromatin status of genes. Indeed, when we looked at those SLOs that are either involved in developmental processes or not (i.e. controlled by gene function), the association between SLOs and the H3K4me3+H3K27me3 bivalent mark (poised status) is consistently significantly higher than expected (56% when controlled for developmental genes (B/T3 in Figure 5) and 33% for non-developmental genes (D/T4 in Figure 5) compared with 17% for genomic background; p = 2.825e-13 and p = 0.007, respectively), showing that the chromatin status in ES cells is also an important factor contributing to the overall TE density of genes.

### Expression data of SUO and SLO genes in ESCs support the ‘open chromatin status’ hypothesis

**Figure 6:**
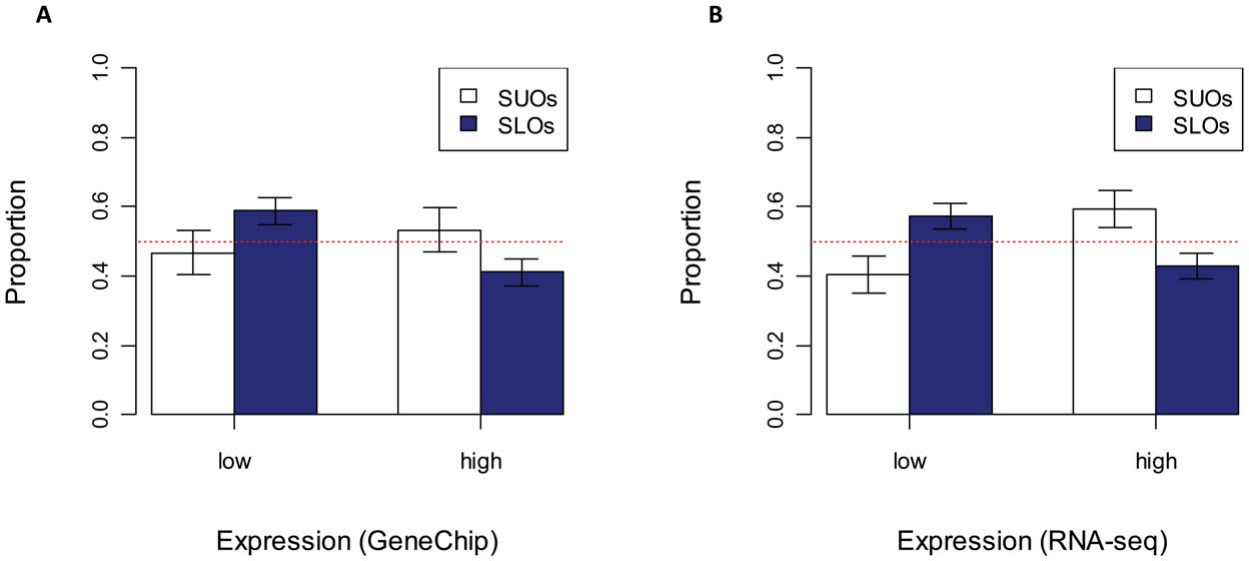
Gene expression data analyses for SUOs and SLOs in mouse ESCs. (A) Gene expression data based on GeneChip technology. (B) Gene expression data based on RNA-seq technology. In both (A) and (B), SUOs and SLOs are divided into ‘low’ and ‘high’ expression genes in mouse ESCs based on the median expression level of all genes > 10 kb. The red dotted line shows the 50% level, which is the proportion taken by lowly/highly expressed genes for all genes > 10 kb in mouse ESCs. Error bars are standard errors derived from the total number of genes (sample size) in each gene set.

If indeed genes with an open chromatin status in ES cells are more prone to heritable TE-insertions, one might expect to see a larger proportion of genes that are highly expressed in ES cells among SUOs. Here we downloaded mouse ES cell expression data previously generated by Mikkelsen et al. [39] and, as expected, when we simply classified SUOs and SLOs into lowly- and highly-expressed genes based on the median genomic transcriptional level in mouse ESCs, we observed that 53% of SUOs are highly expressed in ESCs compared with only 41% for SLOs (Figure 6A). However, while the pattern is clear that SUOs contain a larger proportion of active genes in ESCs than SLOs, the result lacks statistical significance (p = 0.1428). Since chip-based techniques commonly suffer from cross-hybridization (where non-specific binding of probes produces unavoidable experimental noise), we performed the same analysis using a different mouse ESC expression dataset that was recently generated by whole transcriptome sequencing (RNA-seq) [41]. As shown in Figure 6B, we made a similar observation with the RNA-seq data as GeneChip but with a more dramatic difference between the two outlier groups (60% highly expressed genes among SUOs vs. 43% for SLOs; p = 0.0155). The above results suggest that genes with open chromatin status and high expression level in ES cells may be more prone to TE insertions that can be inherited, which could lead to accumulation of TEs in such genes.

Interestingly, a recent study documenting large numbers of somatic L1 retrotransposition events in the human brain showed that such events occur significantly more often in genes expressed in brain compared to random expectations [42]. This study lends support to the view that insertions of at least some TE types are more likely to occur in open chromatin.

### Concluding remarks

The relationship between TEs and host genes has intrigued biologists since the discovery of these mobile DNA sequences in various host genomes. Using the genomic sequences of three distant mammalian species (i.e. human, mouse and cow), we identified “shared outlier” genes bearing highly conserved patterns of unusually high or low TE densities. These outlier genes, denoted as SUOs and SLOs respectively, provide a highly reliable resource for studying TE-gene relations. Here we showed that TE density within genes is not random and, in addition to the widely known G+C content effect, is also associated with other gene properties such as gene function and conservation level. Specifically, SUOs are enriched for metabolic genes and SLOs are enriched for genes involved in developmental processes. Moreover, SUOs show less conservation at the protein sequence level in general, and an expanded analysis involving non-shared outlier genes in each species revealed a disproportionate enrichment of species-specific genes for TE-rich genes (upper outliers). The above findings are also in agreement with previous studies of the association between TE density and gene function [24], [25], [26].

Initial insertional preference, natural selection and genetic drift can all contribute to the current distribution pattern of TEs in host genes. While evaluating initial insertional preference is difficult for many ancient TEs, a limited number of such experiments have shown distinct distribution patterns of *de novo* TE insertions compared to fixed genomic distributions of corresponding TEs [43], [44], suggesting much greater effects of natural selection or genetic drift on fixed elements. Therefore, it is natural to propose that the enrichment of genes involved in developmental processes for SLOs is due to the essential roles that these genes play in the development of the host organism and, as a result, their strong resistance to TE disruption. One can further postulate that the enrichment of metabolic genes among the SUOs could in fact be beneficial since they can potentially contribute to the genetic variation of the host species. However, the comparison of the average TE age shows SUOs contain significantly younger TEs than SLOs, which implies that the high TE density of SUOs could also be due to recent TE insertions, some of which will be lost with time. Furthermore, other results presented here also suggest that the genomic signature of initial insertion site preference may still exist. We found that extreme TE content in introns is clearly associated with the chromatin status and expression level of genes in embryonic stem cells, with upper outlier genes more likely to be active in ES cells and lower outliers the opposite. Given that all heritable TEs are the result of integration events that occurred in either germline cells or early embryos, and if we postulate that TEs are more likely to insert into actively transcribing genes, it is possible that more TEs would accumulate in such genes and less in genes that are inactive in these tissues. Indeed, only a very minor tendency for TEs to insert into actively transcribing genes in the germ line or early embryo could contribute, over evolutionary time, to their resultant densities in genes. In conclusion, our data supports the view that both selection and initial insertion site preference have played a role in the extreme intronic TE densities observed in mammalian genomes.

Finally, while our data provide intriguing insights on TE-gene relationships based on highly reliable gene sets shared in multiple species, caution should be used when applying these results to a broader theme due to the limited numbers of genes studied. On the other hand, our analyses using all (i.e. both shared and non-shared) outlier genes show very similar results, confirming that the findings in this study very likely reflect general characteristics of TE-gene relationships in mammals.

## Materials and Methods

### Selection of source datasets

To obtain the genomic coordinates of TEs in human, mouse and cow, we downloaded the RepeatMasker annotation of TEs from the UCSC Genome Browser (http://genome.ucsc.edu). The versions of genome assemblies that we used are hg18 for human, mm9 for mouse, and bosTau4 for cow. For the annotation of genes, we downloaded RefSeq gene tracks based on the same genome assemblies listed above. Here we defined TE density as the ratio between the total length of TE sequences presented in a given gene and the size of the longest RefSeq isoform of that gene. An analysis of TE density in human genes revealed a normal distribution except a singleton peak at near zero TE density (Figure S1A). After excluding all genes shorter than 10 kb, this peak disappeared (Figure S1B). We applied the same analysis for the mouse and found similar results (data not shown). The above observation indicates that most genes with near zero TE density are just very small genes, which are low in TEs very likely due to their limited size. For this reason, our study only included genes larger than 10 kb. Because we intended to identify outlier genes shared among multiple species, we also confined our data only to genes included in both the NCBI HomoloGene and RefSeq databases.

### Identification of genes with extreme TE densities

In order to identify outlier genes, a cutoff percentile for genes with high/low TE densities was required. Since the selection of this threshold was a balance between the significance of effect and a reasonable outlier population size for the ease of further data analyses, we performed a computational optimization experiment by testing multiple cutoff thresholds of the outlier percentile from 2.5% to 25% with an incremental step of 2.5%. For each percentile tested, we calculated both the total number of SUOs and SLOs derived and the ratio between the theoretical probability by chance and the actual frequency of observing shared outliers among the three species (Table S3). Based on the above optimization results, we chose 10% as the optimized cutoff percentile for outlier genes in each species (The number of genes derived in each step is given in Table S4).

### Normalization of gene size and exon density

In order to eliminate the confounding effects of the size and exon density of the gene, we controlled our outlier selection for both factors. Specifically, we designed a 5×5 factor matrix which is composed of the following two dimensions (Figure S3): the first dimension (horizontal) contains five predefined consecutive ranges of gene size, each including 20% of all genes; the second dimension (vertical) is also made up of five consecutive bins of exon density levels, each containing 20% of all genes within the corresponding range of gene size. In this way, we arranged our gene data into a well-organized lattice in which each unit contains the same number of genes. Based on this factor matrix, we applied our optimized cutoff percentile threshold (i.e. 10%) on each lattice unit to obtain outlier genes with either the highest or the lowest TE density. Finally, all outlier genes identified in each unit (the shaded part at either side of the gene distribution within each unit in Figure S3) were merged into two categories according to whether they are high or low in TEs (i.e. total upper-/lower-outliers). To verify the efficiency of the above normalization strategy, we also applied statistical tests upon the final sets of total upper- and lower-outlier genes and found no significant differences in terms of both gene size and exon density (data not shown).

### Identification of shared-outlier genes

To identify shared outlier genes among the three mammals used in this study, we simply took the HomoloGene IDs (HIDs) of the upper- and lower-outlier genes commonly identified in all three species and defined the result gene set as SUOs and SLOs, respectively. Notably, based on the normalization of gene size and exon density, outlier genes even with a different category of gene size or exon density in each species were still able to be correctly identified as long as they share the same extreme of TE density in all three species.

### Classification of gene conservation levels

To evaluate the relationship between TE density and gene conservation level, we classified all genes in each species into three conservation levels, namely species-specific, mammalian-specific and ancient genes. Based on the HomoloGene Database, here we defined ‘species-specific’ genes as genes found only in one of the three mammalian species used in this study. For ‘mammalian-specific’ genes, we defined them as genes shared between at least two of the three species mentioned above due to the relatively poor annotation of the cow genome. Finally, we defined ‘ancient genes’ as genes present in mammals plus at least one of the following species: zebrafish, fruitfly and yeast.

### Comparison of the average TE age between SUO and SLO genes

To proximately calculate the average TE age for a gene, we collected the RepeatMasker annotation of TEs from the UCSC Genome Browser and obtained the divergence of each TE fragment from the consensus sequence of each TE family. The average age of all TEs in a given gene is calculated with the equation described below:
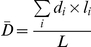
where 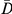 is the average divergence of TE sequences from the consensus (i.e. the age of TEs), *d_i_* is the sequence divergence of each TE fragment in the gene, *l_i_* is the length (in bp) of each TE fragment, and *L* is the total coverage (in bp) of all TEs in the gene. Based on the above method, the average TE age of SUO and SLO genes are calculated and compared using the Wilcoxon Rank Sum test.

### Examination of the tissue specificity of Polr2a binding at SUO/SLO promoters

As suggested by the authors of the Polr2a binding data [38], we defined ‘ubiquitous’ promoter activity based on all five tissues tested as genes with an overall Polr2a binding entropy *H*>2, where *H  =  −∑_1_*_≤t≤N_ *p*_t_log_2_ *p*_t_ (*p* is the relative Polr2a binding strength, *t* is the tissue type, *N* is the total number of tissues tested). Likely, we took genes with *H*≤2 as ‘tissue-specific’ Polr2a binding. The specific tissue type where Polr2a binding shows the strongest activity at the given promoter was identified according to the lowest value of ‘categorical tissue-specificity’ *Q_t_* among all five tissues, where *Q_t_  =  H -* log_2_ *(p_t_)* as described by the above authors.

### Statistical tests

All statistical tests were done in R (version 2.9.2). All p-values described in the text were based on the equality of proportion test (also known as Binomial proportion test) unless specifically noted otherwise.

## Supporting Information

Figure S1 **TE density distribution of human genes.** (A) TE density distribution of all human RefSeq genes. (B) TE density distribution of human RefSeq genes larger than 10 kb.
(TIF)

Figure S2 **The relationship between gene size and exon density in human.** (A) The negative association between gene size and exon density. (B) The linear regression between gene size and the inverse of exon density. *r* is the correlation coefficient.
(TIF)

Figure S3 **Identification of outlier genes by controlling gene size and exon density.** In any given species, all genes ≥ 10 kb were divided into 25 subsets based on both gene size and exon density and were put into a 5 x 5 matrix. For genes in each subset, upper/lower outliers were identified by taking the top or bottom 10% genes with the most extreme TE density. The final set of upper/lower outlier genes is collected by merging the upper/lower outliers from each subset, for which the variations of both gene size and exon density are controlled.
(TIF)

Figure S4 **Chromosomal distribution of SUOs and SLOs in human.** The short red lines along the left side of each chromosome show the chromosomal locations of SLOs. The short blue lines along the right side of each chromosome show the chromosomal locations of SUOs.
(TIF)

Figure S5 **The relationship between the number of SUOs/SLOs on human chromosomes and the chromosome size.** Results for SUOs and SLOs are shown in (A) and (B), respectively. In both (A) and (B), the x-axis shows the genomic coverage of each chromosome in percentage, and the y-axis shows the total number of SUOs/SLOs on a given chromosome.
(TIF)

Figure S6 **Correlation analysis of the TE composition of SUOs between human and mouse.** Results for LINE, SINE, LTR retroelement and DNA transposon are shown as linear regression plot in (A), (B), (C) and (D), respectively. In each plot, each open circle represents an SUO gene and its location is determined by the density of the corresponding TE class of the SUO orthorlogs in the two species. The line across the data points in each plot represents the regression line, and *r* is the correlation coefficient.
(TIF)

Figure S7 **Correlation analyses of G+C content vs. LINE/SINE composition of SUOs in human.** Results for LINE and SINE are shown in (A) and (B), respectively. The x-axis shows the proportion covered by LINEs/SINEs relative to all TEs in each SUO gene. The y-axis shows the average G+C content of human SUOs. The line across the data points in each plot represents the regression line, and *r* is the correlation coefficient.
(TIF)

Figure S8 **Tissue-type composition of tissue-specific outlier genes.** For each gene set, the proportion corresponding to each tissue type is shown in a stacked bar according to the color scheme indicated at the top. The ‘genomic background’ was calculated based on all mouse genes > 10 kb that show strong Polr2a binding in only one tissue.
(TIF)

Figure S9 **Histone marks at promoters of all outlier genes.** The proportions of genes associated with different histone marks are shown for all upper outliers, all lower outliers and the genomic background as side-by-side bars. Error bars are standard errors derived from the total number of genes (sample size) in each gene set.
(TIF)

Table S1 **SLOs and SUOs identified among human, mouse and cow.**
(XLS)

Table S2 **Overrepresentation of GO terms for SUOs and SLOs (BiNGO results).**
(XLS)

Table S3 **The optimization table for outlier threshold selection.**
(XLS)

Table S4 **Number of TUs/genes derived in each step of SUO/SLO calculation.**
(XLS)
