# Supplementary figures and images for: Gene Properties and Chromatin State Influence the Accumulation of Transposable Elements in Genes
Source: PLoS One. 2012 Jan 17;7(1):e30158. doi: 10.1371/journal.pone.0030158 (PMC3260225; doi:10.1371/journal.pone.0030158)

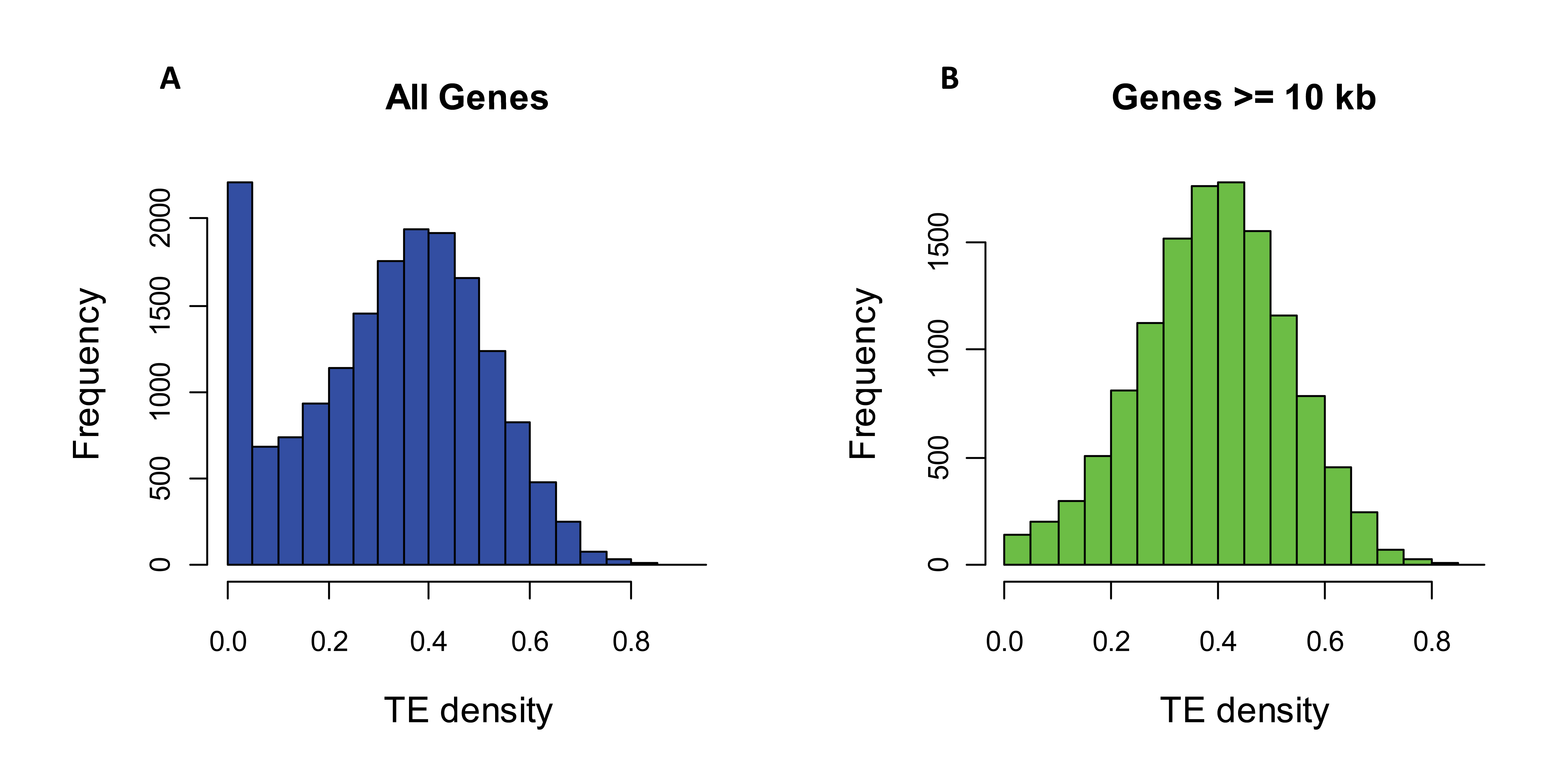

Supplement: Figure S1 — TE density distribution of human genes. (A) TE density distribution of all human RefSeq genes. (B) TE density distribution of human RefSeq genes larger than 10 kb. (TIF) [file pone.0030158.s001.tif]

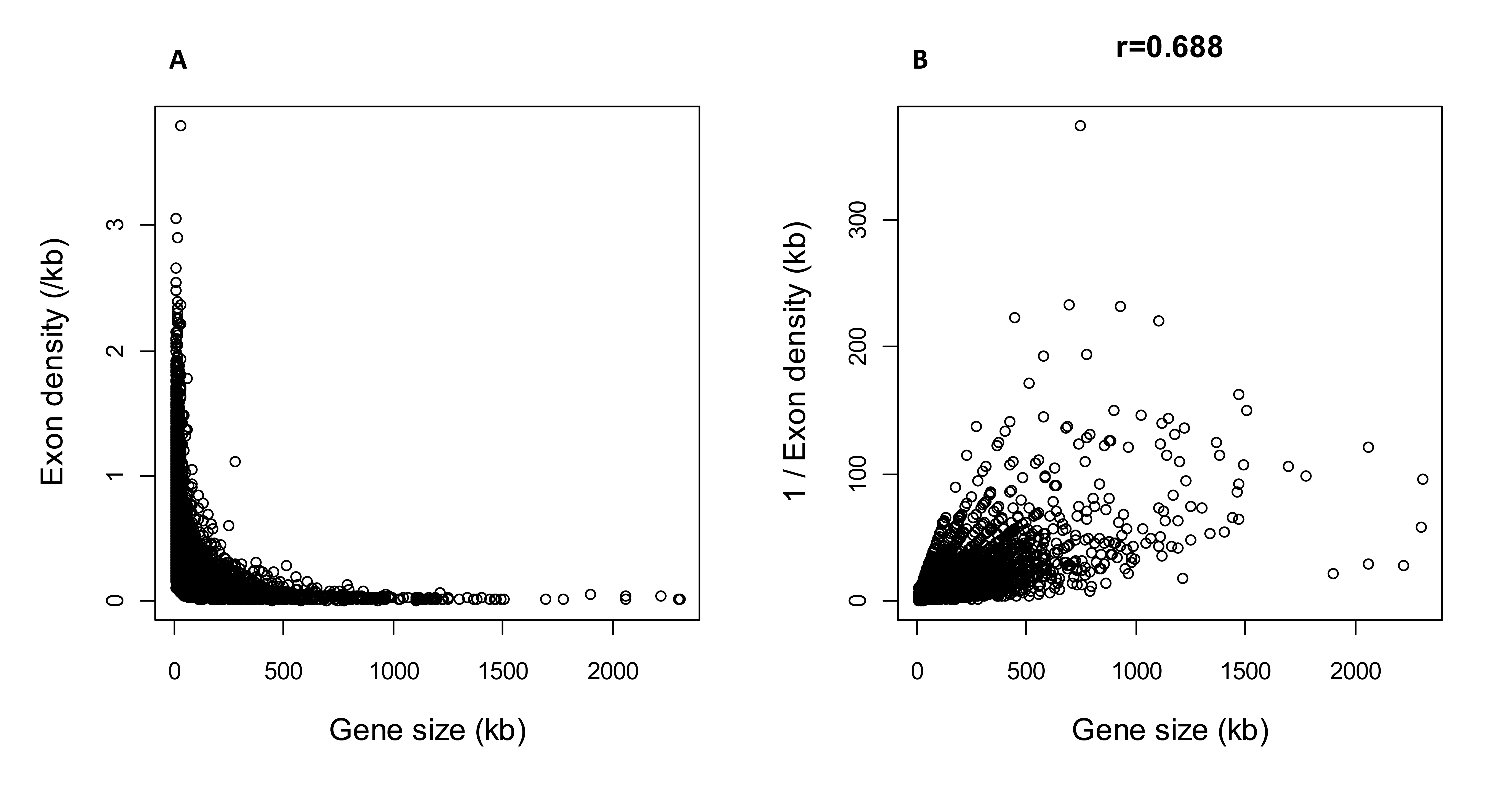

Supplement: Figure S2 — The relationship between gene size and exon density in human. (A) The negative association between gene size and exon density. (B) The linear regression between gene size and the inverse of exon density. r is the correlation coefficient. (TIF) [file pone.0030158.s002.tif]

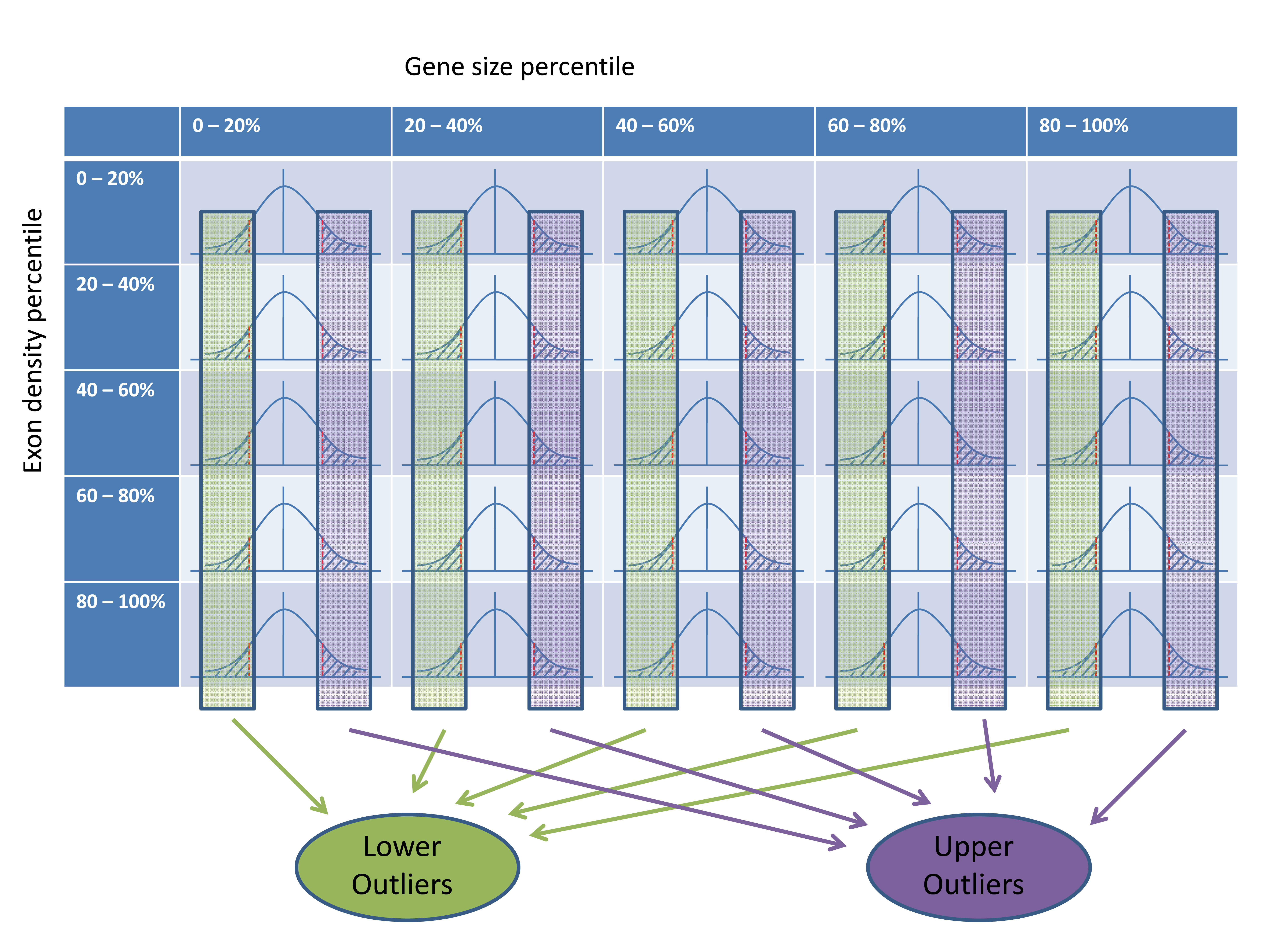

Supplement: Figure S3 — Identification of outlier genes by controlling gene size and exon density. In any given species, all genes ≥ 10 kb were divided into 25 subsets based on both gene size and exon density and were put into a 5 x 5 matrix. For genes in each subset, upper/lower outliers were identified by taking the top or bottom 10% genes with the most extreme TE density. The final set of upper/lower outlier genes is collected by merging the upper/lower outliers from each subset, for which the variations of both gene size and exon density are controlled. (TIF) [file pone.0030158.s003.tif]

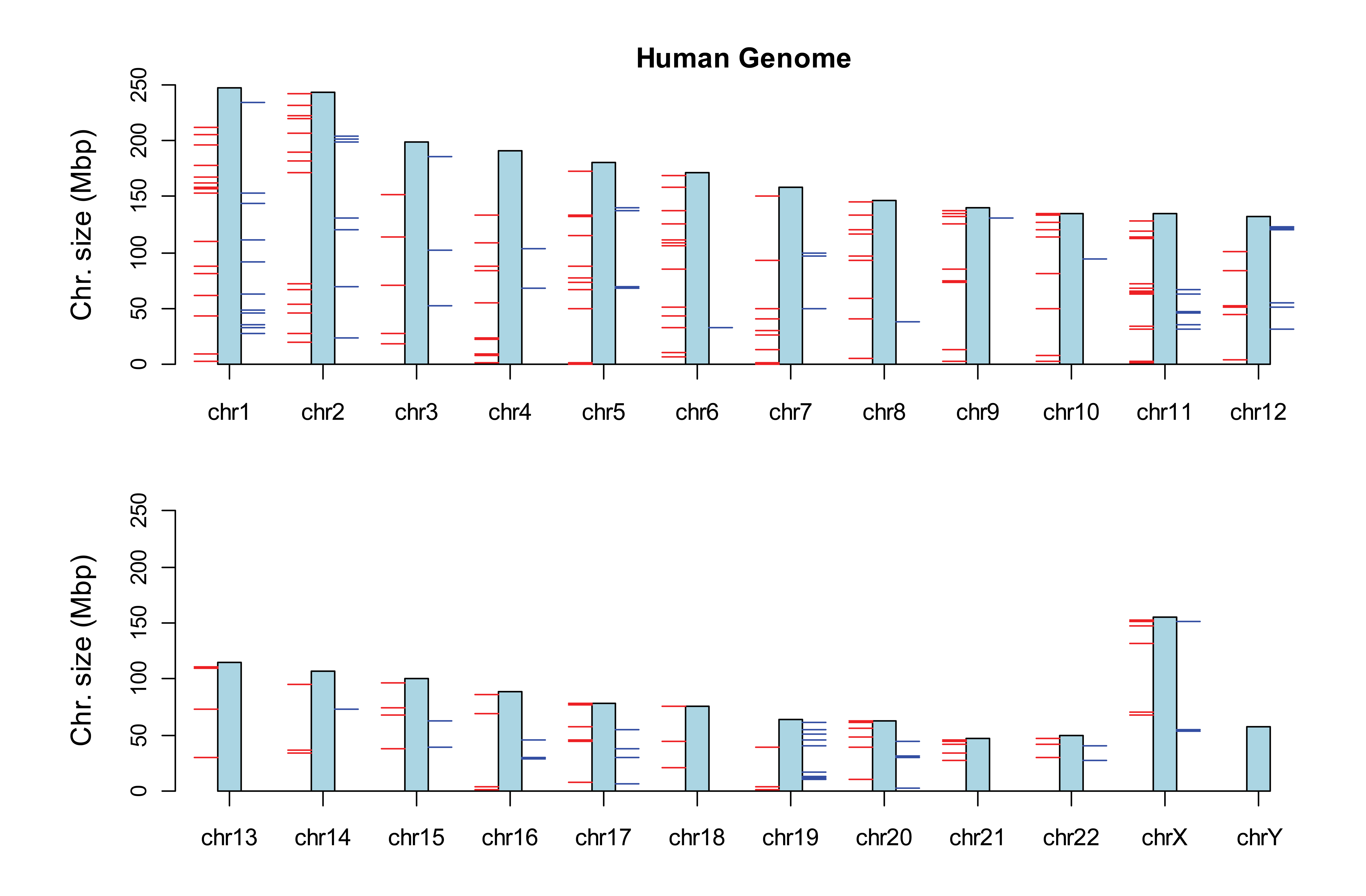

Supplement: Figure S4 — Chromosomal distribution of SUOs and SLOs in human. The short red lines along the left side of each chromosome show the chromosomal locations of SLOs. The short blue lines along the right side of each chromosome show the chromosomal locations of SUOs. (TIF) [file pone.0030158.s004.tif]

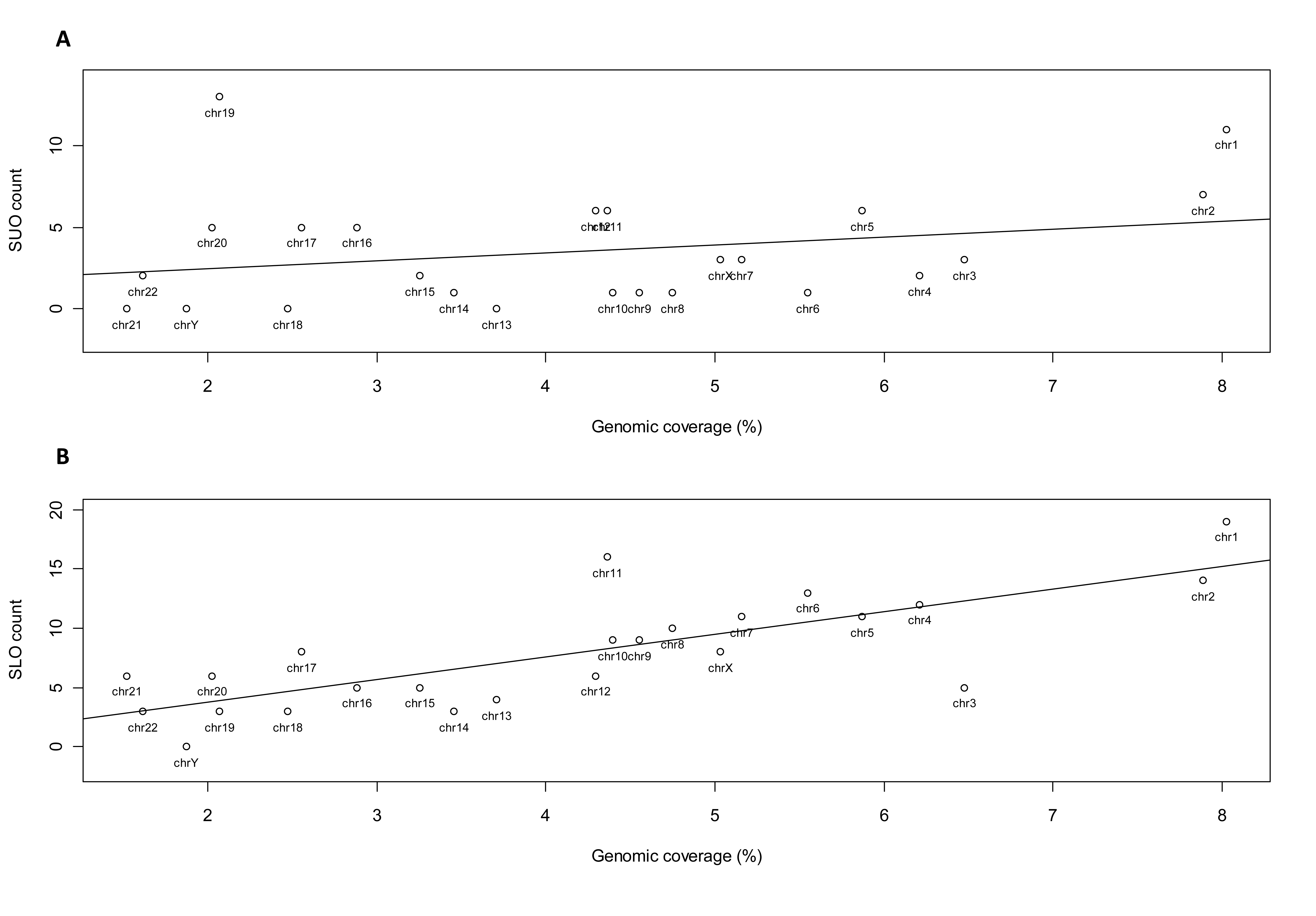

Supplement: Figure S5 — The relationship between the number of SUOs/SLOs on human chromosomes and the chromosome size. Results for SUOs and SLOs are shown in (A) and (B), respectively. In both (A) and (B), the x-axis shows the genomic coverage of each chromosome in percentage, and the y-axis shows the total number of SUOs/SLOs on a given chromosome. (TIF) [file pone.0030158.s005.tif]

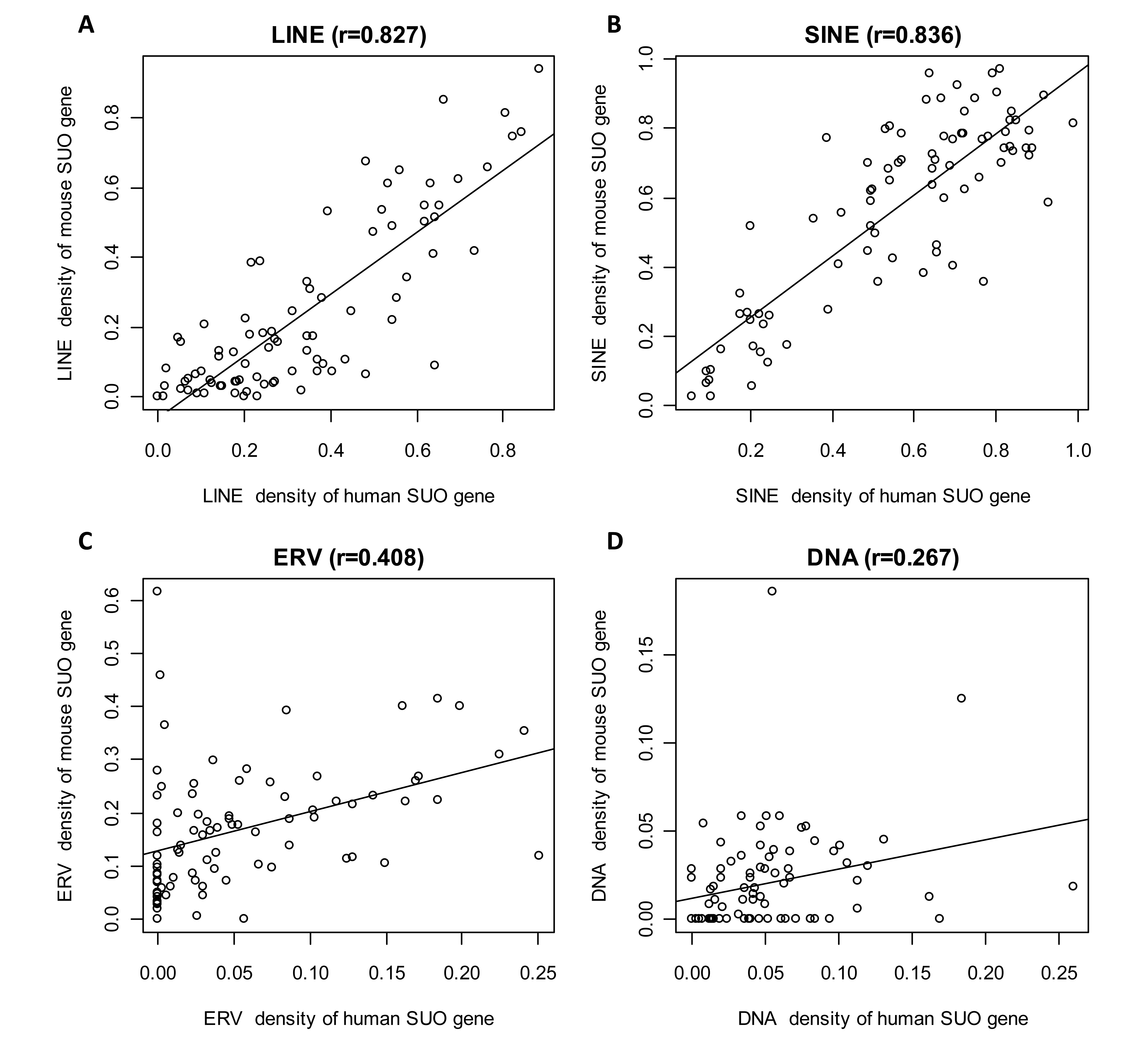

Supplement: Figure S6 — Correlation analysis of the TE composition of SUOs between human and mouse. Results for LINE, SINE, LTR retroelement and DNA transposon are shown as linear regression plot in (A), (B), (C) and (D), respectively. In each plot, each open circle represents an SUO gene and its location is determined by the density of the corresponding TE class of the SUO orthorlogs in the two species. The line across the data points in each plot represents the regression line, and r is the correlation coefficient. (TIF) [file pone.0030158.s006.tif]

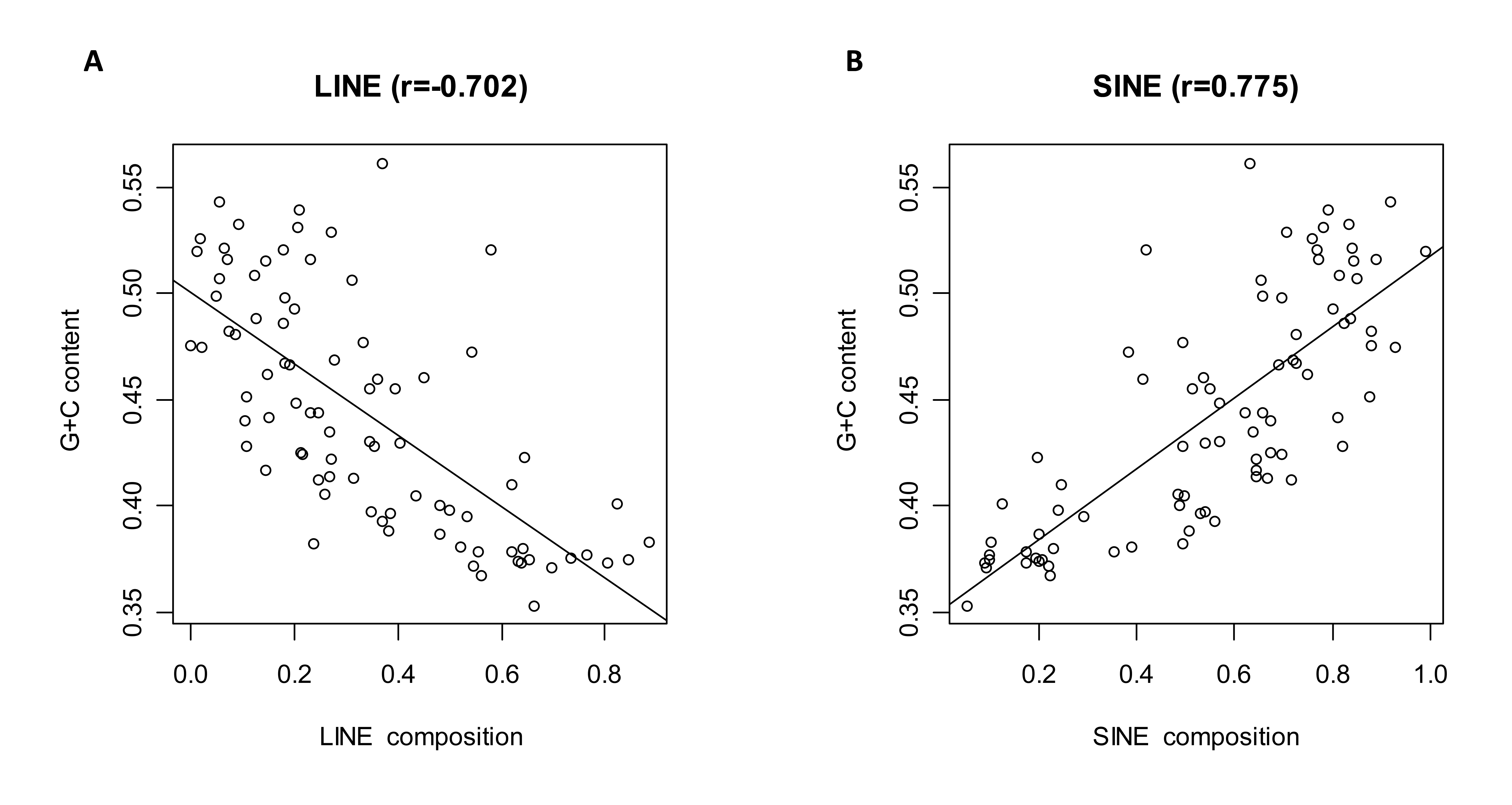

Supplement: Figure S7 — Correlation analyses of G+C content vs. LINE/SINE composition of SUOs in human. Results for LINE and SINE are shown in (A) and (B), respectively. The x-axis shows the proportion covered by LINEs/SINEs relative to all TEs in each SUO gene. The y-axis shows the average G+C content of human SUOs. The line across the data points in each plot represents the regression line, and r is the correlation coefficient. (TIF) [file pone.0030158.s007.tif]

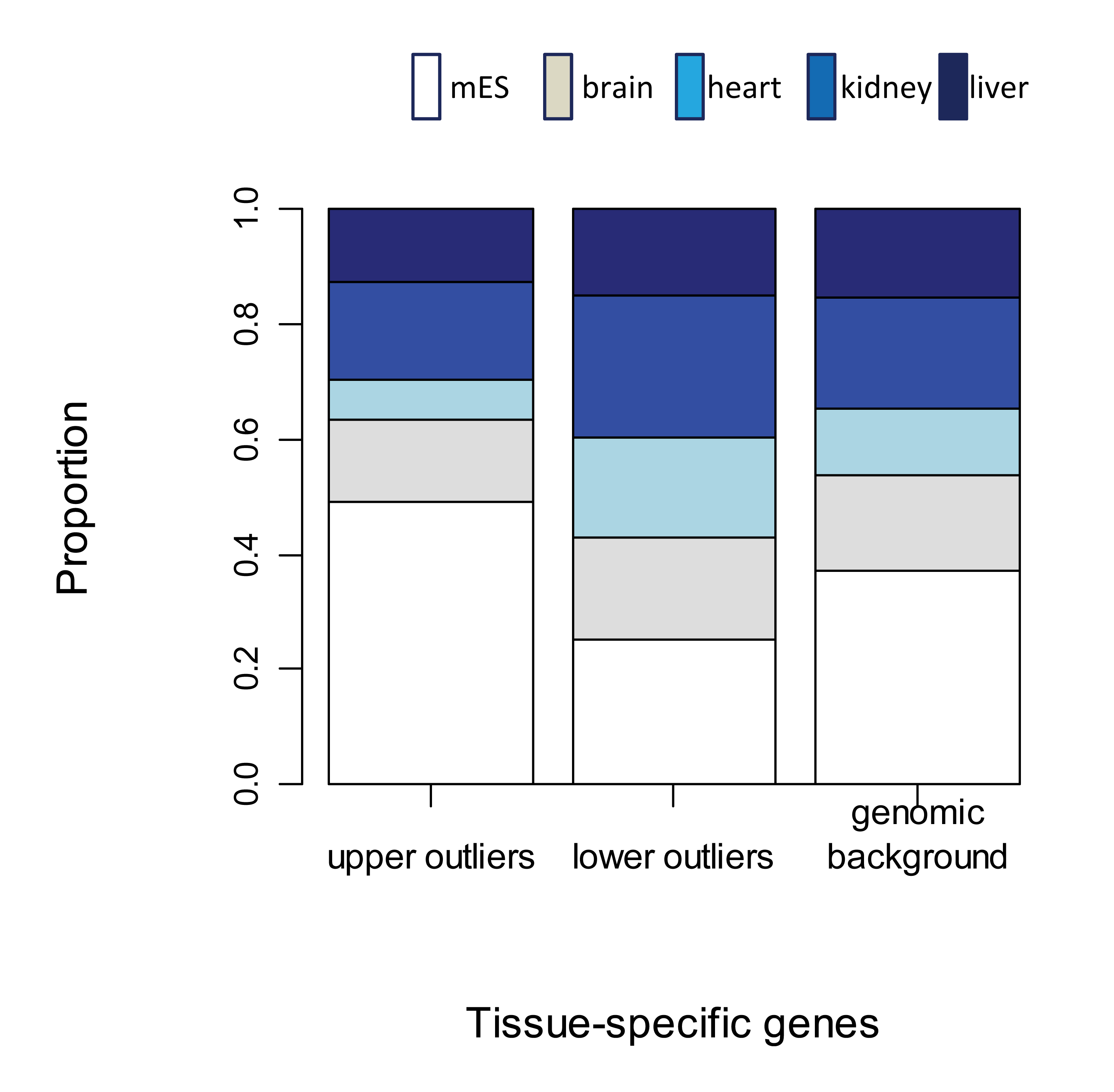

Supplement: Figure S8 — Tissue-type composition of tissue-specific outlier genes. For each gene set, the proportion corresponding to each tissue type is shown in a stacked bar according to the color scheme indicated at the top. The ‘genomic background’ was calculated based on all mouse genes > 10 kb that show strong Polr2a binding in only one tissue. (TIF) [file pone.0030158.s008.tif]

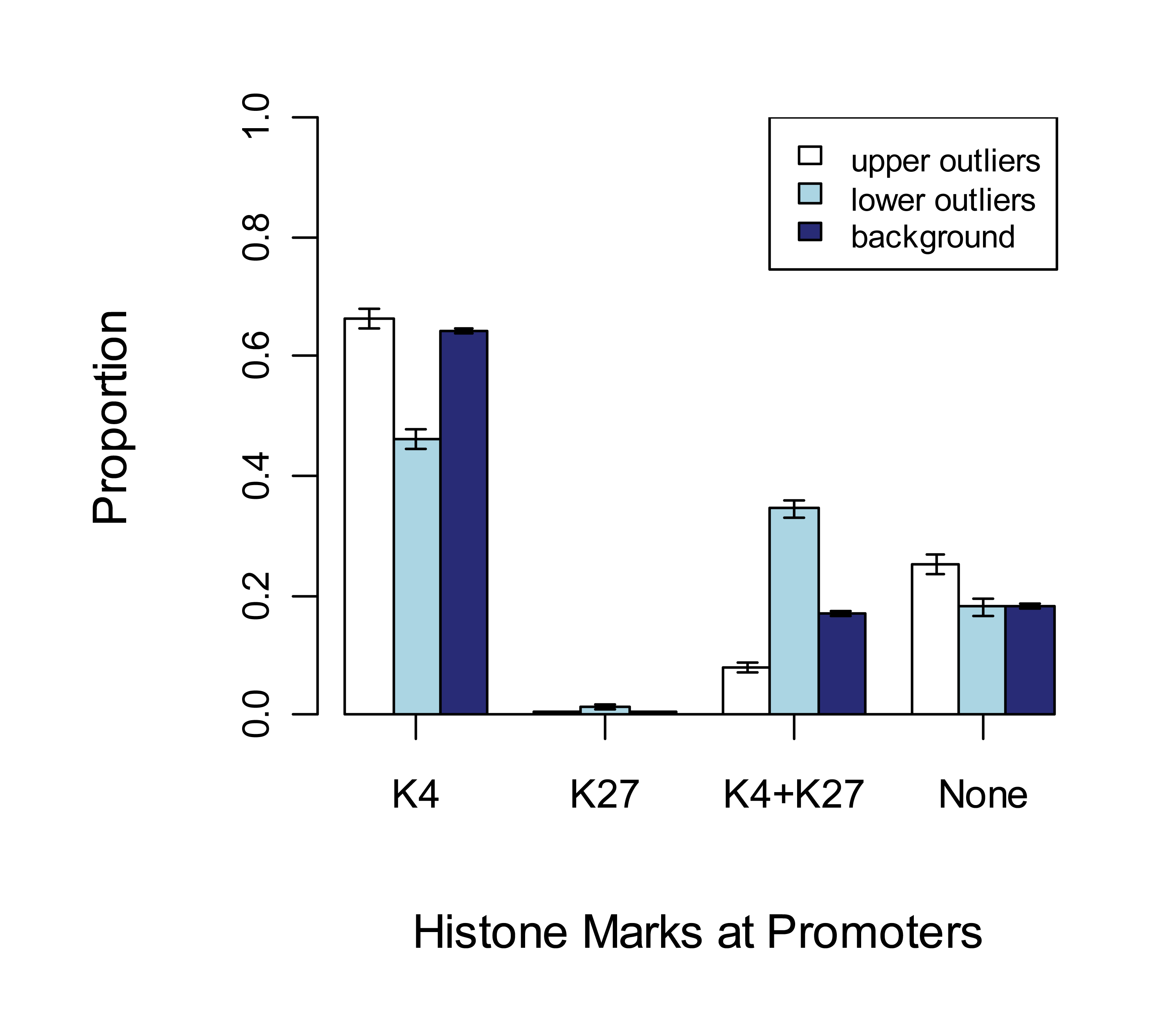

Supplement: Figure S9 — Histone marks at promoters of all outlier genes. The proportions of genes associated with different histone marks are shown for all upper outliers, all lower outliers and the genomic background as side-by-side bars. Error bars are standard errors derived from the total number of genes (sample size) in each gene set. (TIF) [file pone.0030158.s009.tif]
